# Seed Transmission of Tomato Leaf Curl New Delhi Virus from Zucchini Squash in Italy

**DOI:** 10.3390/plants9050563

**Published:** 2020-04-29

**Authors:** Eui-Joon Kil, Thuy Thi Bich Vo, Chairina Fadhila, Phuong Thi Ho, Aamir Lal, Elisa Troiano, Giuseppe Parrella, Sukchan Lee

**Affiliations:** 1 Department of Plant Medicals, Andong National University, Andong 36729, Korea; 2 College of Biotechnology and Bioengineering, Sungkyunkwan University, Suwon 16419, Korea; 3 Istituto per la Protezione Sostenibile delle Piante, CNR, 80055 Portici, NA, Italy

**Keywords:** begomovirus, seed transmission, tomato leaf curl New Delhi virus (ToLCNDV)

## Abstract

*Tomato leaf curl New Delhi virus* (ToLCNDV) is a bipartite begomovirus affecting tomato cultivation on the Indian subcontinent. Recently, however, a new strain of the virus, named ToLCNDV-ES, has spread to Mediterranean countries such as Spain, Italy, and Tunisia, and occurred in Cucurbita crops, causing economic damage. Although ToLCNDV is spread by the sweet potato whitefly (*Bemisia tabaci*), like other begomoviruses, it has not been clear how ToLCNDV suddenly spread from the Indian subcontinent to the Mediterranean region. In 2017, ToLCNDV was diagnosed in young seedlings germinated naturally from fruits fallen in the prior year on a farm located in Giugliano in Campania, Naples, Italy, suggesting a possible role of the seeds in vertical transmission of the virus. Because sweet potato whiteflies were widespread naturally in that region, it was necessary to verify that in an artificial insect vector-free condition. Seeds were harvested from two ToLCNDV-infected zucchini squash cultivars in Naples in 2017 and 2018 to examine whether ToLCNDV can be transmitted from zucchini squash seeds to young plants. Viral DNA was amplified from these seeds and 1- to 3-week-old seedlings germinated from them with a ToLCNDV-specific primer set. According to PCR results, viral contamination was confirmed from all harvested seeds and dissemination was proven from 61.36% of tested seedling samples. Mechanical transmission from seed-borne virus-infected seedlings to healthy zucchini squash plants was also succesful, demonstrating that seedlings from ToLCNDV-infected seeds did act as inoculum. This is the first report demonstrating that ToLCNDV is a seed-transmissible virus in zucchini squash plants in Italy.

## 1. Introduction

*Tomato leaf curl New Delhi virus* (ToLCNDV) is a bipartite begomovirus (genus *Begomovirus*, family *Geminiviridae*) transmitted by the whitefly *Bemisia tabaci* and causes total yield loss in tomato cultivation [1,2]. After the first report of leaf curl disease of tomatoes in India [3], 48 species have been reported as tomato leaf curl disease (ToLCD)-related viruses. ToLCNDV is a representative virus causing ToLCD, which was first reported from tomatoes in India in 1994 [4]. Since then, the virus has spread to other countries on the Indian subcontinent (Bangladesh and Pakistan), to countries in Southeast Asia (Indonesia, Philippines, Sri Lanka, Taiwan, and Thailand), and to the Middle East (Iran) [2,5,6,7,8,9,10,11]. Various isolates have been reported to show genetic variation including mutation and recombination, some of which were defined as new species [12,13]. In recent years, this virus has not been specific to Asia because one of the noble strains of ToLCNDV spread to Spain, named ToLCNDV-ES, which grouped in a different phylogenetic cluster than those reported from Asian countries [14]. Since the first report of ToLCNDV-infected zucchini squash (*Cucurbita pepo*) in southern Spain, introduction of the same viral strain (i.e., ToLCNDV-ES strain) into other Mediterranean countries including, Italy and Tunisia has damaged cucurbit crops [14,15,16,17,18]. ToLCNDV was the first reported bipartite begomovirus in European countries [15].

Zucchini squash (*C. pepo*) is one of the most important horticultural crops in Italy. ToLCNDV has become a representative threat for zucchini squash cultivation [19]. Normally, ToLCNDV-infected zucchini squash show typical symptoms including severe curling, yellow mosaic and vein swelling of young leaves, shortening of internodes, rough skin, and reduced size of fruit [17].

Most of cases, plant viruses can be transmitted by insect vectors and sap of infected plants in fields, which are restricted to horizontal transmission [20]. On the other hand, plant viruses can be transmitted via virus-infected seeds [21], causing their spread beyond the limits of time and place. This is because seed transmission is the way of allowing viruses to transmit through generations, to overcome geographical limitations of horizontal transmission and to survive from longer periods of time than within vectors.

For many years, begomoviruses have been unable to be transmitted by virus-infected seeds and were only transmitted by whitefly *B. tabaci*-mediation, grafting, and artificial inoculation with infectious clones [22]. However, according to recent reports, the following begomoviruses have shown seed-transmissible characteristics: sweet potato leaf curl virus, mung bean yellow mosaic virus, tomato yellow leaf curl virus, bitter gourd yellow mosaic virus, and dolichos yellow mosaic virus [23,24,25,26,27,28,29]. Additionally, seed transmission of the ToLCNDV Indian strain was identified in Chayote in India [30]. ToLCNDV has been a major threat to the cultivation of cucurbit crops in the Mediterranean region [14,15,16,17,18], but there have been no reports on the possibility of seed transmission ToLCNDV in the region. It is very important to check for the possibility of its seed transmission because infected seeds can spread the virus beyond time and space limitations [21]. Thus, our main objective in this study was to determine whether natural infection of zucchini squash plants from two cultivars with ToLCNDV-ES populations under field conditions results in seed transmission of the virus.

## 2. Results and Discussion

### 2.1. ToLCNDV Infection in Zucchini Squash Plants

**Figure 1:**
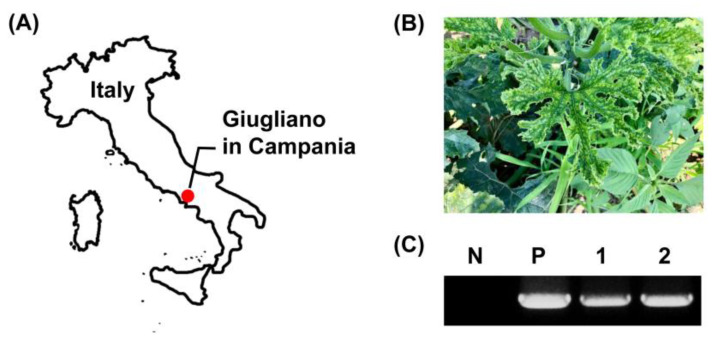
Tomato leaf curl New Delhi virus (ToLCNDV) has been identified in zucchini squash plants in Campania, Italy. (**A**) Location of sampling. (**B**) Symptoms in zucchini squash leaves infected with ToLCNDV. (**C**) PCR analysis was performed with a ToLCNDV-specific primer set from zucchini samples collected from Giugliano in Campania, Italy. Lane N, no template control; lane P, positive control; and lanes 1-2, collected leaf samples from two different symptomatic zucchini squash plants.

As previously reported, ToLCNDV-infected zucchini squash plants are easily observed from farms in Giugliano in Campania, Naples, Italy (Figure 1A,B). In 2017 and 2018, zucchini squash plants with abnormally shaped leaves (Figure 1B) were collected from two different farms. According to PCR results with ToLCNDV-specific primers (Table 1), ToLCNDV was detected from all symptomatic samples from the two farms (Figure 1C), and amplicons verified a previously reported sequence (GenBank accession number MF688670) based on sequencing data (Macrogen, Seoul, Korea).

### 2.2. ToLCNDV Infection of Naturally Germinated Young Seedlings

**Figure 2:**
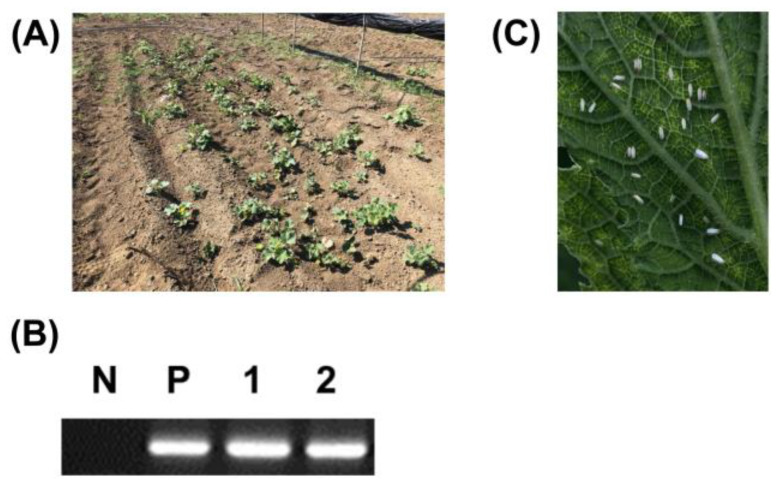
(**A**) Young plants naturally germinated from fallen fruits of tomato leaf curl New Delhi virus (ToLCNDV)-infected plants. (**B**) PCR analysis was performed with a ToLCNDV-specific primer set from young plants. Lane N, no template control; lane P, positive control; and lanes 1-2, seedling samples naturally germinated from fallen fruits of ToLCNDV-infected plants. (**C**) *Bemisia tabaci* in a *Cucurbita* plant cultivation area.

ToLCNDV was first described on tomatoes in India in 1994 [4] and for over 20 years it remained confined to the Asian continent until the first signalling in Europe in 2016, in Spain [14]. To date, it is unknown how ToLCNDV was introduced from India or other Asian countries into the Mediterranean region. The possibility of ToLCNDV seed transmission was suspected based on previous seed transmission reports on other begomoviruses [23,24,25,26,27,28,29]. In the meantime, young seedlings germinated naturally from fruits fallen the prior year were found in a cucurbit farm, where ToLCNDV was detected by chance (Figure 2A). Viral DNA was isolated from five collected seedlings, and ToLCNDV-specific PCR was performed as previously described. PCR amplicons were confirmed from all collected samples (5/5; Figure 2B). However, in that field as in other farms in Naples, sweet potato whiteflies (*B. tabaci*) spread naturally (Figure 2C). We could not be convinced that ToLCNDV in young cucurbit seedlings originated from ToLCNDV-infected seeds. Therefore, it was necessary to verify this result in an isolated insect vector-free condition.

### 2.3. ToLCNDV Contamination of Zucchini Squash Seeds

In order to determine the possibility of seed transmission of ToLCNDV, which was raised before, in the isolated condition that was not exposed to insect vectors or external infection, experiments were conducted using seeds collected from zucchini squash plants of two different cultivars (cvs. Milos and Ortano) infected with ToLCNDV in Naples in 2017 to 2018. After sterilizing, DNA extraction and ToLCNDV-specific PCR were conducted as with 30 seeds of cv. Milos and 10 seeds of cv. Ortano as previously mentioned (see the Section 3.1 *Seeds collection and sample preparation* in Materials and Methods).

All seed samples tested positive for ToLCNDV (Table 2), verifying that all seeds were contaminated with the virus.

### 2.4. ToLCNDV Dissemination of Zucchini Squash Seedlings Germinated from Infected Seeds

**Figure 3:**
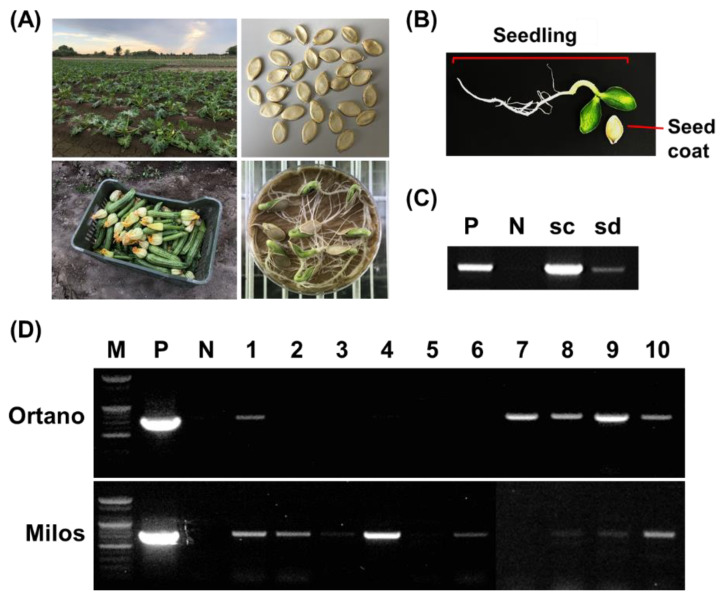
Seed contamination and dissemination analyses from tomato leaf curl New Delhi virus (ToLCNDV)-infected zucchini squash plants. (**A**) Sample preparation from Italian fields. (**B**) Zucchini squash seedling germinated from seed. (**C**) PCR analysis was performed with a ToLCNDV-specific primer set from seed coat (sc) and seedling (sd). (**D**) PCR analysis was performed with a ToLCNDV-specific primer set for young plants germinated from ToLCNDV-infected seeds. Lane M, 100 bp DNA ladder marker (Bioneer, Daejeon, Korea); lane N, no template control; lane P, positive control; lanes 1-2, young plants of zucchini squash cv. Milos (bottom) and cv. Ortano (top).

Germinated seedlings were prepared from seeds from the same fruit as those for which ToLCNDV contamination had been identified. At 1 to 3 weeks after germination, DNA was isolated from seed coats and seedlings separately (Figure 3B). Amplification with ToLCNDV-specific primers was performed as described above, and amplicons were identified in seed coats and seedlings (Figure 3C). Of 44 seedlings from ToLCNDV-infected seeds (28 of cv. Milos and 16 of cv. Ortano), ToLCNDV was detected in 27 seedlings (61.36%; Figure 3D and Table 2). These results confirm that ToLCNDV can be vertically transmitted by infected zucchini squash seeds. While preparing our results, another Indian research groups also demonstrated ToLCNDV seed transmission in Chayote [30], so ours is not the first report on ToLCNDV seed transmission. However, because Indian and Mediterranean isolates of ToLCNDV showed differences (about 10% in nucleotide sequences) [14], our results showed that not only Indian ToLCNDV isolates, but also Mediterranean isolates can be transmitted by infected seeds.

### 2.5. Mechanical Inoculation of Seed-Borne ToLCNDV

**Figure 4:**
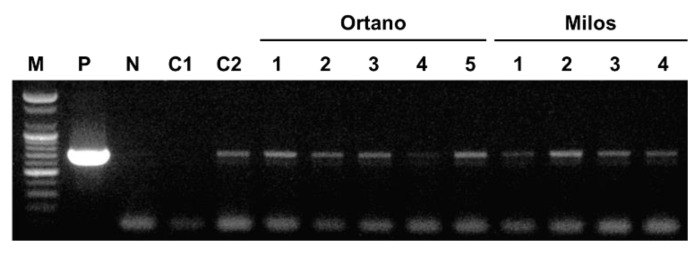
Mechanical inoculation from seed-infected zucchini squash plants to healthy zucchini squash plants. Lane M, 100 bp DNA ladder marker (Bioneer); lane P, positive control; lane N, no template control; lane C1, control from healthy zucchini squash leaves to young, healthy zucchini squash plants; lane C2, control from symptomatic zucchini squash leaves to young, healthy zucchini squash plants.

Unlike most other begomoviruses, ToLCNDV is known for its mechanical transmission [7,31,32]. Such transmission can occur naturally by infected plants in farms, and sap of ToLCNDV-infected leaves can be a good inoculum for artificial inoculation [32,33]. To identify whether seed-borne ToLCNDV can be transmitted from infected zucchini squash seedlings to other healthy plants by sap, homogenized sap was spread on healthy plant leaves. Three weeks after inoculation, from new leaves of five cv. Ortano and four cv. Milos inoculated by sap, all plants were confirmed to be infected with ToLCNDV (Figure 4). This result showed that ToLCNDV from infected zucchini squash seeds can be transmitted to other healthy plants by sap of germinated seedlings.

**Figure 5:**
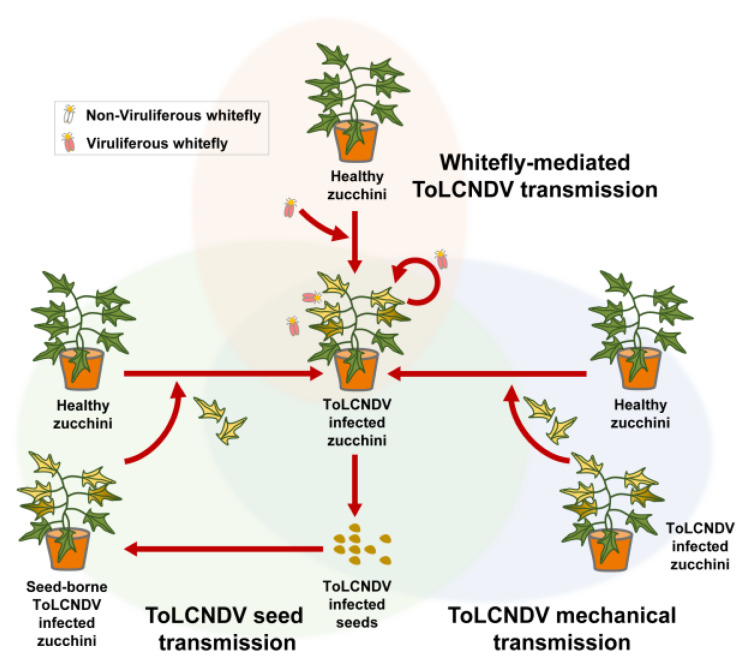
Schematic diagram of the tomato leaf curl New Delhi virus disease cycle according to whitefly-mediated, mechanical, and seed transmission.

ToLCNDV was reported as transmissible by viruliferous whitefly *B. tabaci* [1,2], and mechanical transmission of ToLCNDV was reported in cucurbit [7,31,32]. In this study, we confirmed that ToLCNDV-infected zucchini squash seeds can cause viral occurrence in daughter seedlings. Taken together, these findings suggest a new three methods of ToLCNDV transmission in zucchini squash plants in fields (Figure 5).

According to the results in this study, we suggest a method of ToLCNDV spread to the Mediterranean region, which is geographically separated from the virus origin. That ToLCNDV in the Mediterranean region originated from seedlings germinated from virus-infected seeds harvested in Asia will be difficult to verify. However, it can be proven if it is possible to track the seeds in circulation at the time. Such verification could also highlight efforts to block the spread of new begomoviruses from seed collection sites to Europe.

## 3. Materials and Methods

### 3.1. Seeds Collection and Preparation

Zucchini squash fruits of two different cultivars (cvs. Milos and Ortano) were harvested, and seeds were collected from ToLCNDV-infected plants in Naples in 2017 to 2018. In particular, 30 seeds of cv. Milos (15 seeds from a single fruit in 2017 and 15 from another single fruit in 2018) and 10 seeds of cv. Ortano (from a single fruit in 2017) were chosen randomly from a set of 25–40 seeds obtained per fruit. Collected seeds were washed with distilled water to remove fruit flesh and then dried to store. To prevent contamination during detection, surfaces of collected seeds were sterilized by exposure to 70% ethanol and 10% Clorox (0.4% sodium hypochlorite) (5–10 min), followed by rinsing in sterile distilled water (at least 10 min) [34]. Germinated seedlings were prepared from seeds placed on a wet Wypall^®^ L25 Wipers tissue (Kimberly-Clark, Seoul, Korea) in a Petri dish (SPL Life Sciences, Pocheon, Korea) in a plant bio low chamber (Dasol Scientific, Hwaseong, Korea) at 28 °C (Figure 3A). At 1 to 3 weeks after germination, seedlings were collected for dissemination tests.

### 3.2. DNA Extraction and PCR Analysis for ToLCNDV Detection

Viral genomes were isolated from collected leaf or seed samples with a Viral Gene-spin™ Viral DNA/RNA Extraction Kit (iNtRON Biotechnology, Seongnam, Korea), and virus detection was performed using a T100^TM^ Thermal Cycler (Bio-Rad, Hercules, CA, USA) with a final reaction volume of 20 μL containing ToLCNDV-specific primers which were designed based on the partial sequence of AC1 coding gene previously reported sequence (GenBank MF688670) by Primer-BLAST [35] (Table 1) and 1 × *AccuPower*^®^ PCR Master Mix (Bioneer, Daejeon, Korea). The PCR conditions were as follows: an initial denaturation at 94 °C for 3 min followed by 35 cycles (denaturation at 94 °C for 30 s, annealing at 58 °C for 30 s and an extension at 72 °C for 1 min) and a final extension at 72 °C for 10 min. DNA amplicons were electrophoresed on 1% agarose gels. Each reaction was performed at least three times and figures in results were shown as the sum of numbers from all trials.

### 3.3. Mechanical Inoculation

To confirm viral transmission from seed-borne ToLCNDV-infected zucchini squash seedlings to other healthy plants by sap, 1 g of zucchini squash seedling leaves, which was confirmed to be ToLCNDV-infected by PCR, was collected and ground with a mortar and a pestle in COMAV buffer (50 mM potassium phosphate [pH 8.0], 1% polyvinylpyrrolidone 10, 1% polyethylene glycol 6000, 10 mM 2-mercaptoethanol, and 1% activated charcoal) as described in a previous study [32]. Homogenized sap was spread on healthy plant leaves with carborundum powder and rubbed gently. New leaves from inoculated plants were harvest 3 weeks after inoculation, and DNA preparation and PCR were performed as noted above.

## Figures and Tables

**Table 1:** List of ToLCNDV-specific primers in this study.

Primer Name	Primer Sequence (5′—3′)	Target Size
ToLCNDV_DNA-A_F	GTG ATG TAC TCC CCT GTG CG	738 bp
ToLCNDV_DNA-A_R	ACA AGA CAG ATG CGT TAA AGG TT	

**Table 2:** Infection rates of tomato leaf curl New Delhi virus (ToLCNDV) from seeds of ToLCNDV-infected zucchini squash plants and germinated seedlings.

Plants (Cultivars)	Seed	Seedlings **
Zucchini Squash cv. Milos	30/30 (100) *	16/28 (57.14)
Zucchini Squash cv. Ortano	10/10 (100)	11/16 (68.75)

* Half harvested in 2017 and half in 2018, from single fruits; ** 7–21 days after germination.
